# High-sensitivity Troponin-T levels in reperfused STEMI patients: A comparison with CMR

**DOI:** 10.1186/1532-429X-18-S1-P72

**Published:** 2016-01-27

**Authors:** Manish Ramlall, Steven K White, Heerajnarain Bulluck, Ashraf Hamarneh, John Davies, Derek Yellon, James C Moon, Derek J Hausenloy

**Affiliations:** 1 grid.83440.3b0000000121901201The Hatter Cardiovascular Institute, University College London, London, UK; 2 grid.461344.00000000403741509Basildon and Thurrock Hospitals NHS Foundation Trust, Basildon, UK; 3 grid.139534.90000000103725777Barts Health NHS Trust, London, UK

## Background

In reperfused ST-segment elevation myocardial infarction (STEMI), CMR late gadolinium enhancement (LGE) is the gold-standard for quantifying myocardial infarct (MI) size. Serum cardiac biomarker area-under-the-curve (AUC) is also used (CK-MB, Troponin T and I) and is more widely available than CMR. However, whether acute MI size measured by the 5^th^ generation high-sensitivity Troponin T assays (hs-Trop T) correlates with that by LGE-CMR is not known.

## Methods

Forty-eight patients presenting with an acute STEMI treated by primary angioplasty (PPCI) had CMR and hs-Trop T levels were measured prior to PPCI and at 6, 12 and 24 hours post-PPCI. These 4 time-points were used for the AUC calculation. The assay was a one-step enzyme immunoassay (electro-chemiluminescence based, *Elecsys 2010, Roche, Switzerland*). Of note, it cannot further quantify elevations >10,000 ng/L. CMR was performed on a 1.5-T scanner 3-6 days after PPCI. Acute MI size was quantified by LGE 10 minutes after gadolinium injection (Otsu method) using ImageJ (*National Institutes of Health, Bethesda, Maryland*), which was also used for volume and mass. Microvascular obstruction (+/- haemorrhage) was included in the infarct area. Infarction was expressed in gram mass. Pearson's correlation coefficient and Independent Student's t-test were used.

## Results

Hs-Trop T AUC showed good correlation (r = 0.64, p < 0.0001) with LGE infarct size, as did 12-hour hs-Trop T (r = 0.63, p < 0.0001). There was an inverse correlation between hs-TropT AUC and LV ejection fraction (r = -0.633, p < 0.0001). Hs-TropT AUC levels were also significantly higher (p < 0.0001) in patients with microvascular obstruction (MVO).

## Conclusions

The widely available 5^th^ generation hs-TropT can quantify acute MI size. A single measurement at 12 hours was as good as 24-hour AUC. Elevations in hs-TropT were associated with a lower LV ejection fraction and the presence of MVO.

**Figure 1:**
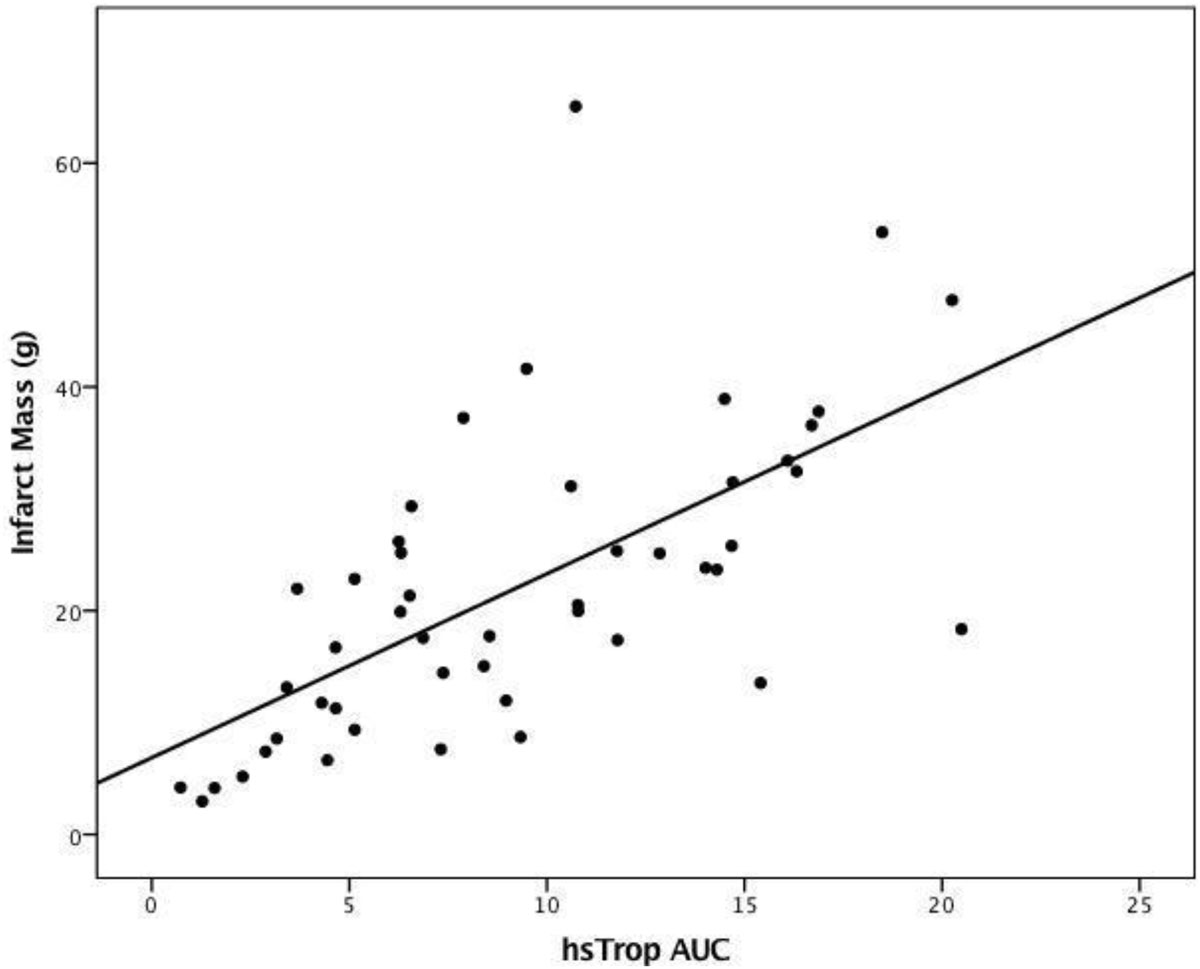
**Scatter-plot of hs-TropT AUC (x10,000 ng/L × hour) measurements against MI size (g)**.

**Figure 2:**
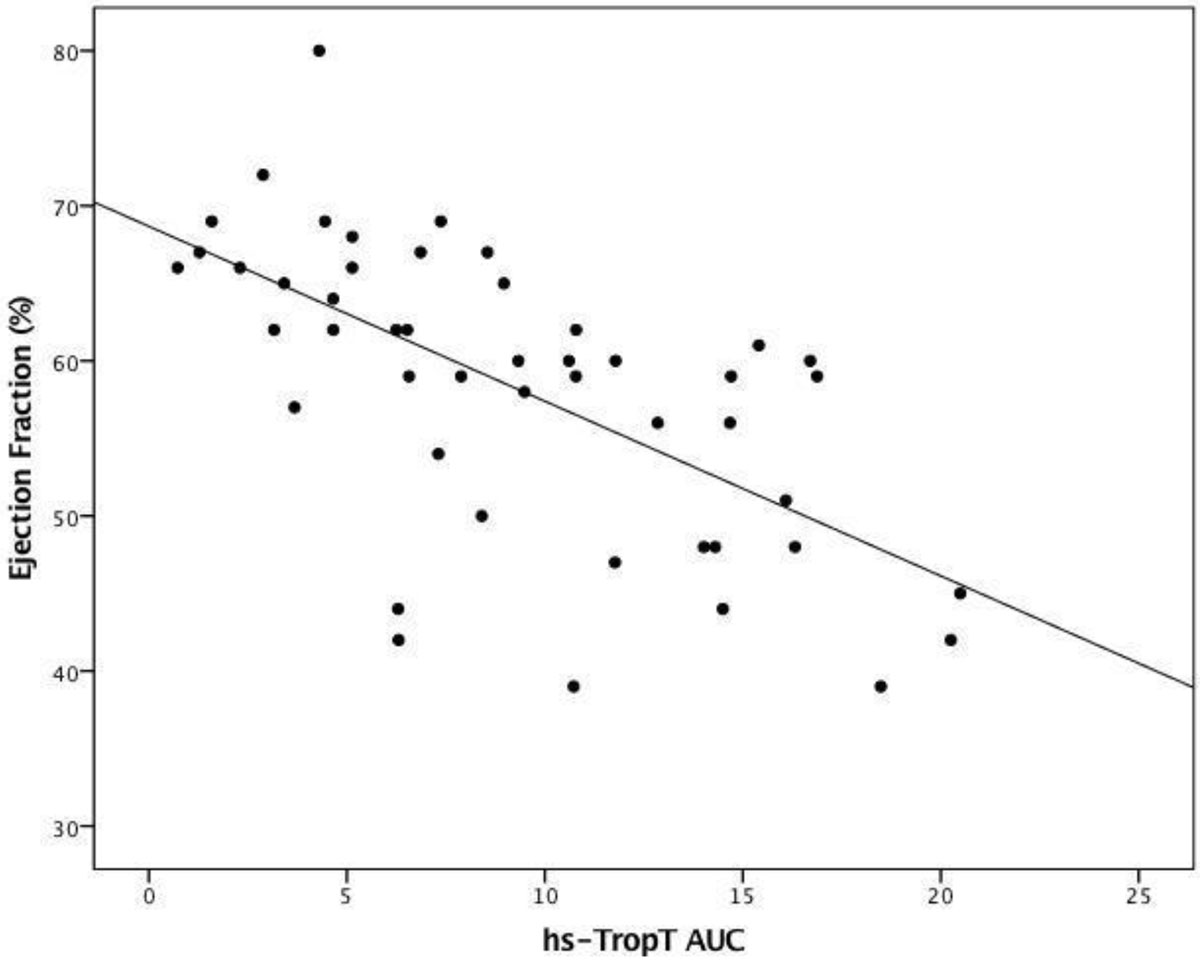
**Scatter-plot of hs-TropT AUC (x10,000 ng/L × hour) measurements against Ejection Fraction (%)**.

